# Impact of Recent SARS-CoV-2 Infection on the Course and Severity of Dengue in Children: A Prospective Observational Study from North India

**DOI:** 10.4269/ajtmh.21-0586

**Published:** 2021-08-02

**Authors:** Namita Ravikumar, Manjinder Singh Randhawa, Karthi Nallasamy, Suresh Kumar Angurana, Mahendra Kumar, Gursimran Kaur Mohi, Radha Kanta Ratho, Muralidharan Jayashree

**Affiliations:** 1 Pediatric Intensive Care and Pulmonology Units, Department of Pediatrics, All India Institute of Medical Sciences, New Delhi, India;; 2 Pediatric Emergency and Intensive Care Units, Department of Pediatrics, Advanced Pediatrics Centre, Postgraduate Institute of Medical Education and Research (PGIMER), Chandigarh, India;; 3 Department of Immunopathology, Postgraduate Institute of Medical Education and Research (PGIMER), Chandigarh, India;; 4 Department of Virology, Postgraduate Institute of Medical Education and Research (PGIMER), Chandigarh, India

## Abstract

In 2020, a considerable overlap occurred between the COVID-19 pandemic and seasonal dengue transmission in India. This study aimed to evaluate the effects of acute or recent infection with SARS-CoV-2 on the course and outcomes of dengue fever in children. We prospectively enrolled 44 children with a clinical and laboratory diagnosis of dengue fever. Assessment of acute and recent SARS-CoV-2 infection was done using reverse transcription–polymerase chain reaction and IgG antibody through ELISA. Children were grouped based on evidence of SARS-CoV-2 exposure and clinical severity, and outcomes were compared. The median age of the study cohort was 96 months (interquartile range [IQR]: 69–129 months). Fever (98%), vomiting (78%), abdominal pain (68%), hepatomegaly (68%), and edema (32%) were the common features. About two-thirds (*N* = 30) had severe dengue; 20 (45%) had dengue shock. Liver dysfunction (58%) and acute kidney injury (25%) were other major organ dysfunctions. Nineteen (43%) children stayed in the pediatric intensive care unit for a median duration of 5 days (IQR: 2–11 days). None had acute SARS-CoV2 infection; however, IgG against SARS-CoV-2 was detected in 15 (34%) cases. Children with recent exposure to SARS-CoV-2 showed a trend toward a lower incidence of acute kidney injury, fewer organ dysfunctions, and a lower frequency of invasive ventilation. Four children (9%) died; none of the deaths were in the SARS-CoV-2–exposed group. The present study exposes preliminary evidence that dengue fever might follow a less severe course in children with recent exposure to SARS-CoV-2 infection. However, it is pertinent to understand the antigenic similarity and cross-protective antibody response between the two viruses and their clinical relevance.

## INTRODUCTION

In 2020, SARS-CoV-2 virus spread rapidly across the globe, resulting in unprecedented health care burden and mortality. Infection in adults is characterized by primarily respiratory system involvement with a wide range of severity.^1^ Children were less severely affected.^2^ However, the relief was short lived as a new multisystem inflammatory syndrome (MIS-C) emerged, almost exclusively in children, a few weeks after exposure to the virus. This was characterized by an exaggerated immune response presenting with fever, rash, conjunctival injection, gastrointestinal symptoms, shock, and multi-organ dysfunction.^3^ Although there were some clinical similarities with Kawasaki disease, it was soon identified as a distinct pathological entity with a different immune-pathological phenotype.^4^ As more research is being carried out, the immunological effects of SARS-CoV-2 infection are emerging. Lymphopenia, alterations in lymphoid cell subsets, and the upregulation of inflammatory cytokines and autoantibodies have been reported in clinical studies.^5^^,^^6^ Altered immunity induced by SARS-CoV-2 has the potential to alter the immune response to other infections. The association between SARS-CoV-2 exposure and other infections at this point is largely unknown. Some preliminary studies reported an increased severity of SARS-CoV-2 infection in patients with pulmonary tuberculosis;^7^ however, limited literature is available regarding its association with dengue.

Dengue fever is an arboviral illness prevalent in southeast Asia during the post-monsoon season. In north India, this season extends from August to November. Dengue is a significant contributor to the outpatient visits and pediatric inpatient admissions during this period.^8^ The peaks of SARS-CoV-2 infections and dengue fever overlapped during the pandemic year of 2020, and health systems were stretched to their limits.^9^^–^^11^ Several countries reported individual case reports of coinfection of SARS-CoV-2 and dengue with variable outcomes and concerns over cross-reactivity between the two antibodies.^12^^,^^13^ From current understanding, the stormy immune response seen with severe dengue may be caused either by dysregulated T-cell response or an exaggerated humoral response due to past infection.^14^ The latter is commonly known as antibody-dependent enhancement. Whether current or recent infection with SARS-CoV-2 is responsible in altering the course of dengue fever is an unanswered but pertinent question. We prospectively enrolled children hospitalized with dengue fever and tested them for active and recent SARS-CoV-2 infection to determine its effect on the clinical course and severity of dengue.

## MATERIALS AND METHODS

This was a prospective observational study conducted at the Pediatric Emergency and Intensive Care Units of a tertiary care teaching hospital in north India between August and December 2020. The protocol was approved by the Institute Ethics Committee (No.INT/IEC/2020/SPL-1523). All consecutive children aged 12 years or younger admitted with clinical illness compatible with dengue were screened for eligibility using a predesigned screening proforma. Those with laboratory-confirmed dengue (detection of NS1 antigen by ELISA and/or IgM anti-dengue antibodies by μ capture ELISA) were enrolled in the study. Children with pre-existing immunodeficiency, inflammatory disorder, or malignancy or those under immunosuppressive therapy were excluded from the study. All enrolled children underwent tests for evidence of acute or recent SARS-CoV-2 infection using Indian Council of Medical Research recommended reverse transcription–polymerase chain reaction assay for SARS-CoV-2 RNA detection or IgG antibodies against SARS-CoV-2 by semiquantitative micro-ELISA (EUROIMMUN kit, Medizinische Labordiagnostika AG 23560 Luebeck, Germany), respectively. The baseline demographic and clinical data were recorded during enrolment. Illness severity was measured using the Pediatric Sequential Organ Failure Assessment (p-SOFA) score and incidence of multiorgan dysfunction syndrome (MODS), defined as per the International Pediatric Sepsis Consensus conference definition.^15^ The worst MODS score, defined as the maximum number of concurrent organ dysfunctions at any time during admission, was recorded. Laboratory parameters at admission, including hemoglobin, platelet count, white blood cell count, serum albumin, liver function tests, and renal function tests, were recorded. Interleukin 6 was measured through cytometric bead array (BD Biosciences, Franklin Lakes, NJ). Details of supportive care, such as mechanical ventilation, vasoactive drug therapy, and hospital outcome, including length of hospital stay, pediatric intensive care unit (PICU) stay, and mortality, were noted.

### Statistical analysis.

Data entry was done in Microsoft excel 2020 (Microsoft, Redmond, WA), and statistical analysis was performed on SPSS software version 22 (SPSS Statistics for Windows, Version 21.0; IBM, Armonk, NY). Categorical variables were described as percentages. Continuous variables were described as mean and standard deviation or median and interquartile range. Proportions were compared between groups using χ^2^ test or Fisher’s exact test, whichever was applicable. Numerical variables were compared between two groups by Student’s *t*-test or Mann-Whitney *U* test, depending upon normality of distribution. A *P* value < 0.05 was considered significant.

## RESULTS

A total of 50 children with clinical illness compatible with dengue were screened, and five were excluded due to confirmation of an alternate diagnosis. One child was excluded from analysis due to sample processing error. Of 44 patients enrolled, 14 tested positive for NS1 antigen, 22 were positive for IgM anti-dengue antibodies, and 8 tested positive for both. The median age of the study population was 96 months (IQR: 60–129 months). Twenty-seven (61%) were boys. The clinical features with severity and outcome are depicted in Table 1. Fever (98%) was the most common presenting feature, followed by vomiting (78%) and abdominal pain (68%). Hepatomegaly (68%) and edema (32%) were the common examination findings. Most patients had profound thrombocytopenia, elevated transaminases, and hypoalbuminemia. The laboratory features are summarized in Table 2. The cohort had a high proportion of severe dengue (*N* = 30; 68%), where two-thirds of them (*N* = 20; 45%) presented with features of shock. Nineteen (43.2%) children required admission to the PICU for a median period of 5 days (IQR: 2–11 days). Organ supportive therapies included invasive ventilation (22.7%) and renal replacement therapy (10%). None of the children was positive for SARS CoV2 RNA by reverse transcription–polymerase chain reaction during hospital admission, whereas IgG antibodies against SARS-CoV-2 were detected in 15 (34%) children, suggesting recent exposure to SARS-CoV-2, although none had symptomatic infection or history of contact with a known case of SARS-CoV-2 infection in the recent past. Age and laboratory markers, including proinflammatory cytokines (IL-6), were comparable between children with and without IgG antibodies against SARS-CoV-2. Interestingly, children with recent SARS-CoV-2 exposure developed fewer organ dysfunctions (acute kidney injury, central nervous system dysfunction) and could be managed with a lower frequency of invasive ventilation. This trend is reflected in the composite organ dysfunction scores (Pediatric Sequential Organ Failure Assessment and MODS scores); however, none of these differences was statistically significant (Table 3). Death was recorded in 4 (9%) children, but no deaths were recorded in those possessing IgG antibodies against SARS-CoV-2.

**Table 1:** Demographic and clinical characteristics of the study population (*N* = 44)

Characteristics	Frequency/Values
Age in months	96 (69–129)
Boys	27 (61)
Clinical features	
Fever	43 (98)
Vomiting	32 (78)
Abdominal pain	30 (68)
Hepatomegaly	30 (68)
Myalgia	17 (39)
Rapid breathing	15 (34)
Edema	14 (32)
Rash	10 (23)
Severity	
Dengue with warning signs	14 (32)
Severe Dengue	30 (68)
Dengue shock syndrome	20 (45)
Compensated shock	13 (29.5)
Hypotensive shock	7 (15.9)
Need for inotrope/vasoactive drug therapy	17 (38.6)
Duration of inotrope/vasoactive drug therapy in hours	60 (24–115)
Encephalopathy	9 (20.9)
Liver dysfunction	25 (58.1)
Acute kidney injury	11 (25)
KDIGO	
Stage 1	5 (11.3)
Stage 2	2 (4.5)
Stage 3	4 (9)
Renal replacement therapy	4 (9.1)
Invasive ventilation	10 (22.7)
p-SOFA score at admission	5 (3–7)
Worst p-SOFA score	5 (3–7)
PICU admission	19 (43)
Length of PICU stay in days	5 (2–11)
Length of hospital stay in days	4 (3–6)
Deaths	4 (9.1)

IQR = interquartile range; KDIGO = Kidney Disease: Improving Global Outcomes; PICU = pediatric intensive care unit; p-SOFA = Pediatric Sequential Organ Function Assessment. Values expressed are in numbers (%) or median (interquartile range).

**Table 2:** Laboratory parameters in study patients

Parameters	Positive (%)/values
Dengue diagnosis	
NS1 antigen	14 (32)
IgM antibody	22 (50)
Both NS1 and IgM	8 (18)
SARS-CoV-2 infection	
RT-PCR	0
IgG antibody	15 (34)
Hemoglobin at admission (g/dL)	12 (10.7–14)
Total leukocyte count at admission (×10^9^ cells/L)	8.45 (4.87–11.65)
Platelet count at admission (×10^9^ cells/L)	26 (16–47)
Lowest platelet count (×10^9^ cells/L)	21 (12–43)
Aspartate transaminase (IU/L)	607 (161.5–1,470)
Alanine transaminase (IU/L)	235.5 (66–616.75)
Albumin (g/dL)	2.5 (2.03–3.13)
IL-6 (pg/mL)	20.7 (11.1–67.4)

RT-PCR = reverse transcription polymerase chain reaction. Values expressed are in numbers (%) or median (interquartile range).

**Table 3:** Comparison between SARS-CoV-2 exposed and unexposed groups

	SARS-CoV-2 exposed (*N* = 15)	SARS-CoV-2 unexposed (*N* = 29)	*P* value
Age in months	84 (48–120)	96 (66–132)	0.40
Hemoglobin at admission (g/dL)	12.6 (11.1–14.2)	12 (10.4–13.8)	0.54
Total leukocyte count at admission (×10^9^ cells/L)	8.7 (3.9–11.83)	8.2 (4.9–11.6)	0.91
Platelet count at admission (×10^9^ cells/L)	29 (2–46)	24 (13–5)	0.34
Lowest platelet count (×10^9^ cells/L)	25 (19–46)	19 (1–40.5)	0.15
Aspartate transaminase (IU/L)	472.5 (72.5–1,930)	607 (165–1,431)	0.64
Alanine transaminase (IU/L)	178.5 (50.75–907.75)	281.5 (68–580.25)	0.78
Albumin (g/dL)	2.5 (2–3.28)	2.5 (2–2.8)	0.57
Interleukin-6 (pg/mL)	20.7 (10.2–67.4)	22.3 (11.4–84.2)	0.77
Severe dengue	10 (67)	22 (76)	0.52
Shock	8 (53)	12 (41)	0.45
Encephalopathy	1 (6.7)	8 (28.6)	0.13
Liver dysfunction	7 (46)	18 (64)	0.26
Acute kidney injury	1 (6.7)	10 (34)	0.06
Stage 1	0 (0)	5 (17.9)	
Stage 2	1 (6.7)	1 (3.6)	
Stage 3	0 (0)	4 (14.3)	
Renal replacement therapy	1 (6.7)	3 (10.3)	1.00
Invasive ventilation	1 (6.7)	9 (31)	0.13
p-SOFA score at admission	3 (3–6)	5.5 (3–8.5)	0.25
Worst p-SOFA score	4 (3–7)	6 (3–10.5)	0.15
Organ dysfunctions at admission	1 (1–3)	2 (1–4)	0.08
Worst MODS score	2 (1–3)	3 (1–4.5)	0.07
Length of PICU stay, days	4.5 (2–9.75)	5 (2.5–11)	0.75
Length of hospital stay, days	4 (2–6)	4 (3–8.5)	0.68
Deaths	0	4 (13.7)	0.28

MODS = multiorgan dysfunction syndrome; p-SOFA = Pediatric Sequential Organ Function Assessment. Values are expressed in numbers (%) or median (interquartile range).

## DISCUSSION

**Figure 1.**
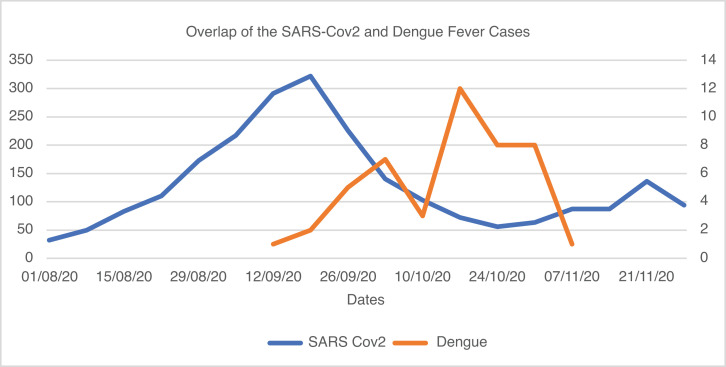
Nearly overlapping peaks of SARS-CoV-2 cases in the region where the study was conducted and children with dengue presenting to the study site. This figure appears in color at www.ajtmh.org.

In this prospective observational study, it was observed that one-third of all hospitalized children with dengue during the 2020 outbreak had recent exposure to SARS-CoV-2 infection. The clinical outcomes, including mortality, length of PICU, and length of hospital stay, were not different between children with and without SARS-CoV-2 exposure. There was, however, a trend toward a lower frequency of acute kidney injury and in the number of organ dysfunctions in children with recent exposure to SARS-CoV-2. A considerable overlap between the COVID-19 pandemic and seasonal dengue outbreak was noted during 2020 in India. The peak cases of COVID-19 in the Chandigarh region were reported between August 15 and October 15, 2020, coinciding with dengue transmission (Figure 1). This is in concurrence with reports from Brazil and other regions where both viruses peaked simultaneously, causing co-epidemics.^16^ Several studies from different parts of the world have reported a decline in the incidence of respiratory viral infections during 2020, possibly due to widely following COVID-appropriate behavior, including the use of masks, limiting outdoor activities, and closure of schools.^17^^–^^20^ In fact, a similar reduction was observed in feco-orally transmitted infections.^19^ Dengue, however, is a mosquito-borne arboviral disease with a seasonal incidence. Asian tiger mosquitoes, *Aedes albopictus* and *Aedes aegypti*, are the principal vectors of its transmission. The effects of the ongoing COVID-19 pandemic and alterations in social structure on dengue are not very clear. The National Vector Borne Disease Control Program of India reported a sharp decline of 75% (39,419 versus 157,315) in dengue incidence during 2020 as compared with 2019. The possible reasons for this reduction could be lower transmission of the vector, less exposure due to COVID-19 lockdown, and, potentially, a disparity in the reported data possibly due to underdiagnosis or underreporting because of increased attention to the COVID-19 pandemic.^21^ In an earlier study conducted in 2019 at our center, 41 children with dengue were enrolled, a figure comparable to the current study.^22^

The clinical presentation of dengue in this study was very similar to previous cohorts. The majority of patients presented with fever and one or more warning signs. At the outset, it may be difficult to differentiate between dengue and COVID-19, particularly MIS-C, in children due to overlapping clinical and laboratory features. Fever, headache, myalgia, abdominal pain, and vomiting are some of the shared clinical features in dengue and MIS-C. Leukopenia, thrombocytopenia, and elevated transaminases are common laboratory abnormalities. In addition, a false‐positive serology for dengue has been noted in patients with confirmed COVID‐19.^23^^,^^24^ In a study in adults, Joubert et al^25^ have observed that nonsevere dengue cases were more symptomatic than mild to moderate COVID-19. Body aches, headache, and retro-orbital pain were the presentations in dengue, whereas anosmia and delayed presentation (> 3 days post onset) were suggestive of COVID-19. No literature is available to establish whether SARS-CoV-2 exposure alters the clinical presentation or course of dengue. In our experience, the clustering of cases during the post-monsoon season, the presence of third spacing, hemoconcentration, and profound thrombocytopenia were some of the distinguishing features noted in dengue.

We noted a trend toward a higher incidence of acute kidney injury and a greater number of organ dysfunctions in children not exposed to SARS-CoV-2; however, due to the small sample size, we were not able to draw meaningful conclusions. Researchers from Brazil reported that the intensity of SARS-CoV-2 epidemic, in terms of number, severity, and rapidity of spread of infections, was lower in areas where a high prevalence of dengue fever was reported in the previous season.^26^ The biological plausibility of this hypothesis has been elucidated by molecular docking studies, which showed that antibodies to dengue virus bound to antigens on the SARS-CoV-2 virus.^27^ This indicates the existence of antigen mimicry between the two viruses and a possibility of cross-reactivity between the antibodies.^28^ Some authors expressed uncertainty over the possible course of dengue fever in patients with past exposure to SARS-CoV-2. The dilemma was whether the cross-reactive antibodies would provide partial protection against severe dengue infection or lead to increased severity due to the well-known phenomenon of antibody-dependent enhancement seen in dengue.^27^ We hypothesize that the presence of antibodies to SARS-CoV-2 might decrease the severity of dengue. Larger multicentric clinical studies or population data from dengue endemic regions might help in understanding the existence of the cross-reactive, cross-protective association between these two single-stranded, positively coiled +SS RNA viruses.

The strength of the present study lies in its novel attempt to assess the effect of recent SARS-CoV-2 exposure on dengue infection in children. Both these viruses are of significant public health concerns in the region. However, the study has a limitation of small sample size.

The present study exposes preliminary evidence that dengue fever might follow a less severe course in children with recent exposure to SARS-CoV-2 infection. However, it is pertinent to understand the antigenic similarity and cross-protective antibody response between the two viruses and their clinical relevance.
